# Tetramethylpyrazine attenuates periorbital allodynia and neuroinflammation in a model of traumatic brain injury

**DOI:** 10.1186/s12950-017-0161-8

**Published:** 2017-06-08

**Authors:** Zhijing Wang, Qi Wang, Cuijie Wang, Xiuzhen Xu, Hongmei Yu

**Affiliations:** 1 Operating theatre, Cangzhou Central Hospital Brain Branch, Cangzhou City, Hebei Province 061000 China; 2 Department of Anesthesiology, Cangzhou Central Hospital Brain Branch, Cangzhou City, Hebei Province 061000 China; 3 The Fourth Department of Neurosurgery, Cangzhou Central Hospital Brain Branch, Cangzhou City, Hebei Province 061000 China

**Keywords:** Traumatic brain injury, Tetramethylpyrazine, Pain, Neuroinflammation

## Abstract

**Background:**

Traumatic brain injury (TBI) is a public health issue. As the major complaint in 51% of TBI patients, chronic pain is an important aspect in TBI treatment. Tetramethylpyrazine (TMP) is an important compound in *Ligustrazine*, an analgesic drug in traditional Chinese medicine, but its potential in relieving pain symptom in TBI has not been tested. We established a TBI mouse model with controlled cortical impact (CCI), and measured periorbital hypersensitivity with von Frey monofilaments. We examined activated microglia and astrocytes and the levels of substance P (SP) and inducible isoform of nitric oxide synthase (iNOS) with immunohistochemistry, measured mRNA and protein levels of proinflammatory cytokines with qPCR and enzyme-linked immunosorbent assay, respectively. Western blot was employed to detect molecules in NF-κB signaling pathway.

**Results:**

TMP significantly attenuated periorbital hypersensitivity in TBI mice. Within 3 days after CCI, TMP attenuated activation of microglia and astrocytes, levels of SP, iNOS, and CGRP in trigeminal pathway, and levels of proinflammatory cytokines (including IL-6, TNF-α, IL-12). In isolated microglia, TMP attenuated the effects of lipopolysaccharide on the phosphorylation of cytoplasmic IKKα/β and IKB-α, and levels of nucleic p65.

**Conclusion:**

TMP reversed periorbital hypersensitivity by limiting neuroinflammation at the primary stage of TBI, and could be a promising drug for pain treatment in TBI.

## Background

Traumatic brain injury (TBI) affects approximately 10 million people annually in the world, and becomes a public health issue [1]. But there are few satisfactory treatments available [2] because TBI patients have a wide-range of neurological manifestations, including chronic pain, mood disorders, sleep disturbance, and so on [1, 3], and underlying mechanisms of each remain unclear. Systematic clinical studies reveal that chronic pain is the major complaint in 51% of TBI patients [3, 4]. Therefore, pain management might be an important aspect for TBI treatment.

Tetramethylpyrazine (TMP) is an alkaloid compound extracted from *Ligustrazine*, an herb having been used as an analgesic drug in traditional Chinese medicine [5]. A series of randomized controlled clinical trials demonstrate satisfactory efficacy of *Ligustrazine* as an adjunctive therapeutic drug for angina pectoris [6]. This benefit might be relevant to its anti-nociceptive property because TMP inhibits myocardial ischemic nociceptive signaling [7]. Accumulating experimental studies reveal that TMP is also effective to relieve some other types of pain states. For instance, local injection of TMP attenuates acute pain induced by local injection of ATP, formalin, or PGE2 [5]. Chronic administration of TMP significantly increases mechanical withdrawal threshold and thermal withdrawal latency in chronic constriction injury mice [8, 9], and in mice suffering from burns [10].

Electrophysiological and pharmacological evidence reveals that TMP inhibits ionotropic purinergic receptors (P2X), which are involved in various types of pain [7]. TMP attenuated ATP-evoked depolarizing currents in dorsal root ganglion neurons [5, 10, 11]. The mice experiencing burns exhibit lower mechanical withdrawal threshold and shorter thermal withdrawal latency, meanwhile, the P2X receptors in dorsal root ganglia are upregulated; in contrast, TMP treatment dramatically attenuates the upregulation of P2X receptors and improves pain hypersensitivity [10]. Therefore, P2X receptors in primary sensory neurons might be major targets for TMP in relieving pain.

In the present study, we utilized controlled cortical impact (CCI) as an established TBI mouse models [12] to assess the effects of TMP. We found that TMP significantly alleviated mechanical allodynia in CCI mice. Meanwhile, TMP reversed the adverse effects of CCI on several neurochemical parameters, inflammatory reactions, and NF-κB signaling pathway. The present study revealed that TMP may be a promising therapeutic drug for pain symptoms in TBI.

## Methods

### Animals

All animal care and experimental protocols were approved by Animal Care and Research Committee in University of Cangzhou Central Hospital Brain Branch. The 8-week-old male C57BL/6 mice, weighed 22–24 g, were used in this study. The mice were housed less than 4 per cage in an environment with stable temperature (22 ± 2 °C), humidity (45–75%), and 12 h light/dark cycle. The food and water were provided to mice ad libitum. The mice were randomly assigned into 3 groups, and were respectively subject to craniotomy as sham control, controlled cortical impact (CCI), and CCI + TMP.

### Traumatic brain injury

A traumatic brain injury (TBI) model was produced with CCI as described previously [13]. C57BL/6 mice were anesthetized with isoflurane (4% for induction; 1-2% for maintenance), and body temperature was maintained with a heating pad. A 4-mm craniotomy was performed to expose somatosensory cortex in the right hemisphere. CCI was induced by placing an electromagnetic stereotaxic impactor (a smooth blunt stainless-steal tip) (Leica Biosystems Richmond, formerly MyNeuroLab, Richmond, IL), perpendicular to the cortical surface and operating it at 1.0 mm depth and 3.0 m/s velocity (100 ms contact time) for 20 min. This protocol has been reported to produce mild TBI symptoms, such as, pain states, motor deficit, anxiety, and neurodegeneration [2, 13–15]. Then, the bone flap was sealed with a permanent cyanoacrylate-based fast-acting adhesive, and the skin was sutured. Sham mice were subject to craniotomy and the same period of anesthesia. The surgery durations in mice were controlled approximately 20 ± 5 min. Buprenorphine (0.05 mg/kg) was subcutaneously administered to facilitate postoperative recovery.

### Periorbital mechanical threshold test

Mice were restrained in a 10 cm long plastic tube with an inner diameter of 3.5 cm, and were allowed to acclimatize to the restrainer for 5-10 min. The von Frey monofilaments (North Coast Medical, Inc., Morgan Hill, CA, USA) with calibrated bending forces (0.008–2.0 g) were used to determine mechanical thresholds. The von Frey monofilaments were applied to make firm perpendicular contact with the skin in periorbital region on the right and left side of the face. Positive responses included vigorous forepaw stroke of face, head withdrawal from the stimulus, or head shaking.

Each mouse was stimulated 10 times bilaterally with each filament, and mechanical thresholds were defined as the von Frey force that caused >50% positive responses.

The von Frey filament assay is commonly used in pain research to detect pain threshold of hind paws in most studies, while that of orofacial region in much less cases. Additionally, TBI is reported to induce trigeminal pain [13], periorbital allodynia [16], head, neck, and body pain [15], and forepaw allodynia [15], etc. To understand the impact of TBI on trigeminal system, and to test the therapeutic potential of TMP in TBI treatment, we employed Periorbital mechanical threshold test.

### Immunohistochemistry

Immunohistochemistry was performed to measure the levels of substance P (SP), inducible isoform of nitric oxide synthase (iNOS), and the activation of microglial, macrophages and astrocytes in somatosensory cortex and medullary trigeminal zones. 1, 3, 7, and 14 days after surgery, the mice were euthanized with overdosed sodium pentobarbital, underwent cardiac perfusion with heparinized saline and then 4% paraformaldehyde. Brains were removed and post-fixed in phosphate buffer solution (PBS) containing 4% paraformaldehyde for 2 h. After then, the brains were transferred to 30% sucrose in PBS until sinking. Brain blocks containing forebrain (Bregma +1.10 to −2.5 mm) and brainstem (Bregma −5.40 to −8.24 mm) were sectioned into 10 μm thick coronal slices with a cryostat at −24 °C, and air-dried overnight.

Tissues were washed and incubated in 10% normal goat serum in 0.3% Triton X-100 for 1 h, followed by PBS containing the following primary antibodies: (1) rabbit anti-SP (1: 250; Millipore catalog no. AB1566); (2) rabbit anti-iNOS (1:100; Enzo Life Sciences, Farmingdale, NY); (3) rabbit anti-ionized calcium-binding adaptor molecule-1 (Iba-1), a cytoplasmic peptide selectively expressed in monocytes and microglia (1: 250; Wako Pure Chemical Industries, Osaka, Japan); (4) rabbit anti-glial fibrillary acidic protein (GFAP) (1:500; Millipore no. AB1540) specific to astrocytes. After 24 h incubation with the primary antibody at 4 °C, tissues were washed 3 times (10 min each), and then incubated with goat anti-rabbit secondary antibodies, conjugated with DyLight 488 or 549 (Jackson ImmunoResearch, West Grove, PA, USA) for 2 h at room temperature. The slices processed with the same procedure except for primary antibody incubation were used as control to exclude false-positive staining.

The immune-stained slices were imaged under an Olympus BX-51 upright fluorescent microscope (Center Valley, PA, USA), and the images were captured through a camera controlled by SPOT Advanced (Sterling Heights, MI, USA). ImageJ (version 1.43 J, NIH, Bethesda, MD, USA) was applied to analyze the images, including quantification of the percentages of SP- and iNOS-positive neurons. The averaged data were from 3 sections per mouse brain.

Microglia and astrocytes were counted with a Nikon NIU microscope (Nikon, Tokyo, Japan) at a 20× objective for three sampling frames (100 μm^2^ each frame) from two serial sections for each animal (six total counting frames). All six frames per section were averaged and are reported as the mean ± standard deviation.

### Primary cell cultures

New born mice (postnatal day 1–2, P1-2) were euthanized. Their brains were removed, were cut into small pieces (<1 mm^3^), and were digested with 0.25% trypsin. After the digestion was terminated with BSA, cells were isolated by filtering with a 40-μm mesh, and then were cultured in complete DMEM medium. Medium was changed 24 h after plating, and then once every 3 days. 2 weeks later, we collected microglia from the cultures by intensive washing and shaking (250 rpm) for 1 h at 37 °C. The collected microglia were labeled for CD11b, and the purity of the samples was determined by flow cytometry. Only the samples with a purity >97% were included for analysis.

### Cell viability assay

The 3-(4, 5-dimethylthiazol-2-yl)-2, 5-diphenyltetrazolium bromide (MTT) assay was used to measure cell viability. In brief, microglia at a density of 1 × 10^4^ cells/well were treated with different concentrations (1, 5, 10, 15 and 20 μM) of TMP for 24 h. Then 20 μl MTT solution (5 mg/ml in PBS; Sigma, St. Louis, MO, USA) was added to each well and incubated for 4 h. The supernatant was removed, and the crystals were dissolved by adding dimethyl sulfoxide (100 μl/well; Sigma, St. Louis, MO, USA). The absorbance at 490 nm was determined using an automatic enzyme-linked immunosorbent assay reader (Bio-Rad, Hercules, CA, USA).

### Quantitative polymerase chain reaction (qPCR)

The qPCR was performed according to the method described previously [17]. Microglia were isolated from mouse brain. Total RNA was extracted, and then complementary DNA libraries were generated using a commercially available kit (Qiagen, Valencia, CA, USA). The qPCR master mixture was purchased from Qiagen. After adding cDNA samples and pairs of primers in the master mixture, qPCRs were run for 40 cycles in a thermocycler (BioRad, Hercules, CA, USA). The primers used in this study were listed in Table 1. The relative expression level for each gene was calculated using the 2^-∆∆Ct^ method [18] and all PCR values were normalized to those of β-actin.

**Table 1:** q-PCR-primers

Gene	Forward	Reverse
IL-6	CTTCGGTCCAGTTGCCTTCT	TGGAATCTTCTCCTGGGGGT
TNF-α	TGGGGAGTGTGAGGGGTATC	TGCACCTTCTGTCTCGGTTT
IL12a	TTCGCTTTCATTTTGGGCCG	ATCAGCTTCTCGGTGACACG
IL12b	AGAACTTGCAGCTGAAGCCA	CCTGGACCTGAACGCAGAAT
Arg1	GGAAGTGAACCCATCCCTGG	CGAGCAAGTCCGAAACAAGC
β-actin	CTACAATGAGCTGCGTGTGG	AAGGAAGGCTGGAAGAGTGC

### Enzyme-linked immuno-absorbent assay (ELISA)

Brains were collected from mice 3 days after surgery. Cytokines, including IL-6, TNF-α and IL-12, in each sample were measured with sandwich ELISA [19]. Briefly, antibodies of these cytokines were non-covalently adsorbed onto 24-well plastic plates. After washing out free antibodies, the sample was applied to the plate, and incubated for 2 h at room temperature. After the plate was washed with PBS for 3 times (10 min each), biotin-conjugated anti-cytokine antibodies were added to bind to each cytokine, followed by adding horse radish peroxidase-labeled avidin or streptavidin. ABC HRP kit was used for generating color, and the optical density was measured with a spectrophotometer (Bio-Rad).

### Luciferase assay

The HEK293 cells (2 × 10^5^) were seeded on 24-well plates and transfected with IL-6 promoter reporter plasmid by standard calcium phosphate precipitation [20]. In the same experiment, empty control plasmid was added to ensure that each transfection receives the same amount of total DNA. To normalize for transfection efficiency, 1 μg pRL-TK promoter Renilla luciferase reporter plasmid was added to each transfection. Luciferase assays were performed with a dual-specific luciferase assay kit (Promega, Madison, WI, USA). Firefly luciferase activities were normalized based on Renilla luciferase activities.

### Chemicals

TMP was obtained from Wuxi Seventh Reagent Factory in China. TMP was dissolved and diluted in 0.9% saline. Other chemicals were purchased from Sigma-Aldrich.

### Statistical analysis

All results were expressed by mean ± S.E.M. Differences between treatment groups were analyzed by Student’s t-test or, where appropriate, ANOVA followed by Dunnett’s post-hoc test for multiple comparisons. *p* value <0.05 was considered to be statistically significant.

## Result

### TMP attenuated mechanical allodynia in periorbital region of CCI mice

**Fig. 1:**
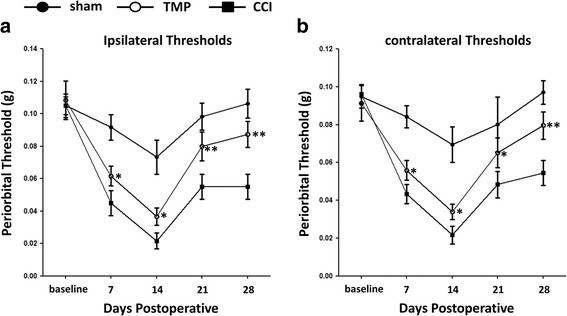
TMP relieved mechanical allodynia in periorbital region in CCI mice. Controlled cortical impact (CCI) was performed on the surface of the somatosensory cortex in the right hemisphere. Periorbital thresholds in both ipsilateral (**a**) and (**b**) contralateral sides were measured with von Frey filament assay. Sham (solid circle), CCI (solid square) and CCI + 4 mM TMP (open circle) mice. *n* = 6 in each group. Data are presented as mean ± SEM values and representative of at least three independent experiments. * *P* < 0.05; ** *P* < 0.01, compared with CCI mice

We measured mechanical threshold in periorbital regions of mice before surgery and 1, 2, 3, and 4 weeks after CCI and sham surgery (Fig. 1a, b). We observed that CCI mice showed mechanical allodynia in periorbital regions on both sides of their faces, in comparison with sham mice. The periorbital thresholds reached lowest levels 2 weeks after surgery. In Sham mice, mechanical threshold decreased at week 2, while recovered to the normal levels one week later (Fig. 1a, b). To test whether TMP attenuates periorbital hypersensitivity in CCI mice, we subcutaneously administered 0.4 mM (200 μL) TMP to these mice. As illustrated in Fig. 1a, and b, in CCI mice, TMP reduced the deterioration of the hypersensitivity in the first 2 weeks, but accelerated its recovery after week 2. These data indicated that TMP has therapeutic potential to relieve periorbital hypersensitivity in CCI mice.

### Effects of TMP on trigeminal pathway in CCI mice

**Fig. 2:**
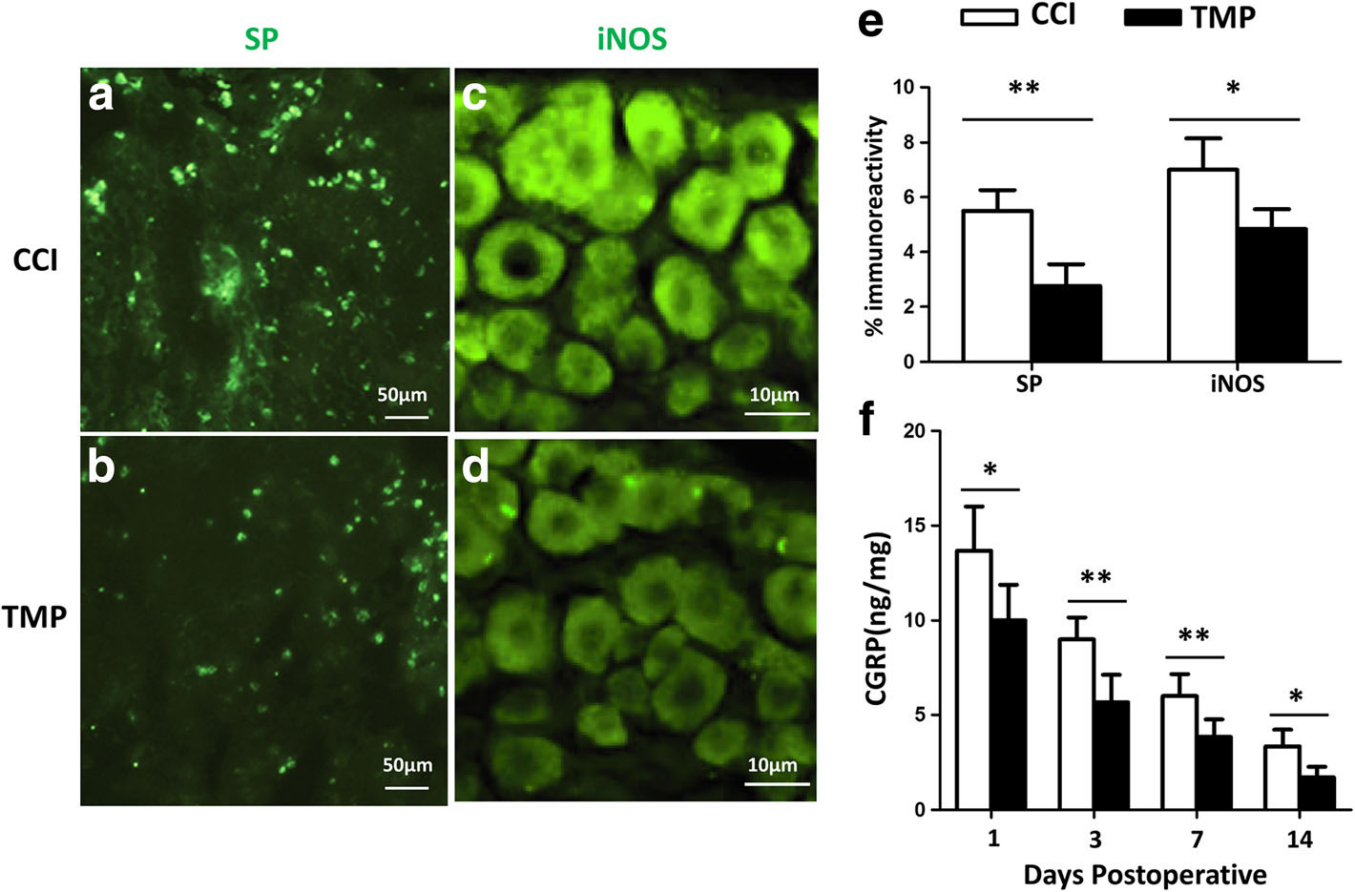
TMP downregulated SP, iNOS, and CGRP in trigeminal pathway of CCI mice. (**a**,**b**) SP immunofluorescent images taken from spinal trigeminal nucleus of CCI mice without (CCI) and with (TMP) TMP (4 mM) treatment 3 days after surgery. (**c**, **d**) Immunofluorescent images showing iNOS-positive trigeminal ganglion neurons in CCI mice without (CCI) and with (TMP) 4 mM TMP 3 days after surgery; (**e**) Percentages of SP- and iNOS- positive neurons in the spinal trigeminal nucleus and trigeminal ganglia in CCI mice with (TMP) and without (CCI) treatment of 4 mM TMP 3 days after surgery; (**f**) Enzyme-linked immunosorbent assay was performed to quantify CGRP levels in the trigeminal nucleus caudalis in CCI mice with (TMP) and without (CCI) daily treatment of 4 mM TMP 1, 3, 7, 14 days after surgery. *n* = 6 in each group. Data in (**e**, **f**) were obtained from at least three independent experiments, and are presented as mean ± SEM. *P < 0.05; **P < 0.01

To address neurochemical alterations accompanied with periorbital hypersensitivity, we examined the levels of SP, iNOS, and calcitonin gene-related peptide (CGRP) in trigeminal pathway. Our immunohistochemistry data showed that TMP administration dramatically reduced the expression of SP in spinal trigeminal nucleus (Fig. 2a, b), and iNOS in trigeminal ganglion (Fig. 2c, d) in CCI mice 3 days after surgery. Meanwhile, TMP also reduced the percentage of SP-positive neurons in spinal trigeminal nucleus, and that of iNOS-positive neurons in trigeminal ganglion (Fig. 2c, d). We measured CGRP in trigeminal nucleus caudalis with ELISA (Fig. 2f). The data showed that CGRP gradually diminished in CCI mice, 1, 3, 7, and 14 days after surgery, and TMP caused a significant reduction in CGRP at each of these time points, compared with CCI mice without TMP treatment. Therefore, we concluded that TMP reduced the expression of SP, iNOS, and CGRP in trigeminal pathway in CCI mice.

### TMP reduced the activation of macrophage/microglia and astrocyte in CCI mice

**Fig. 3:**
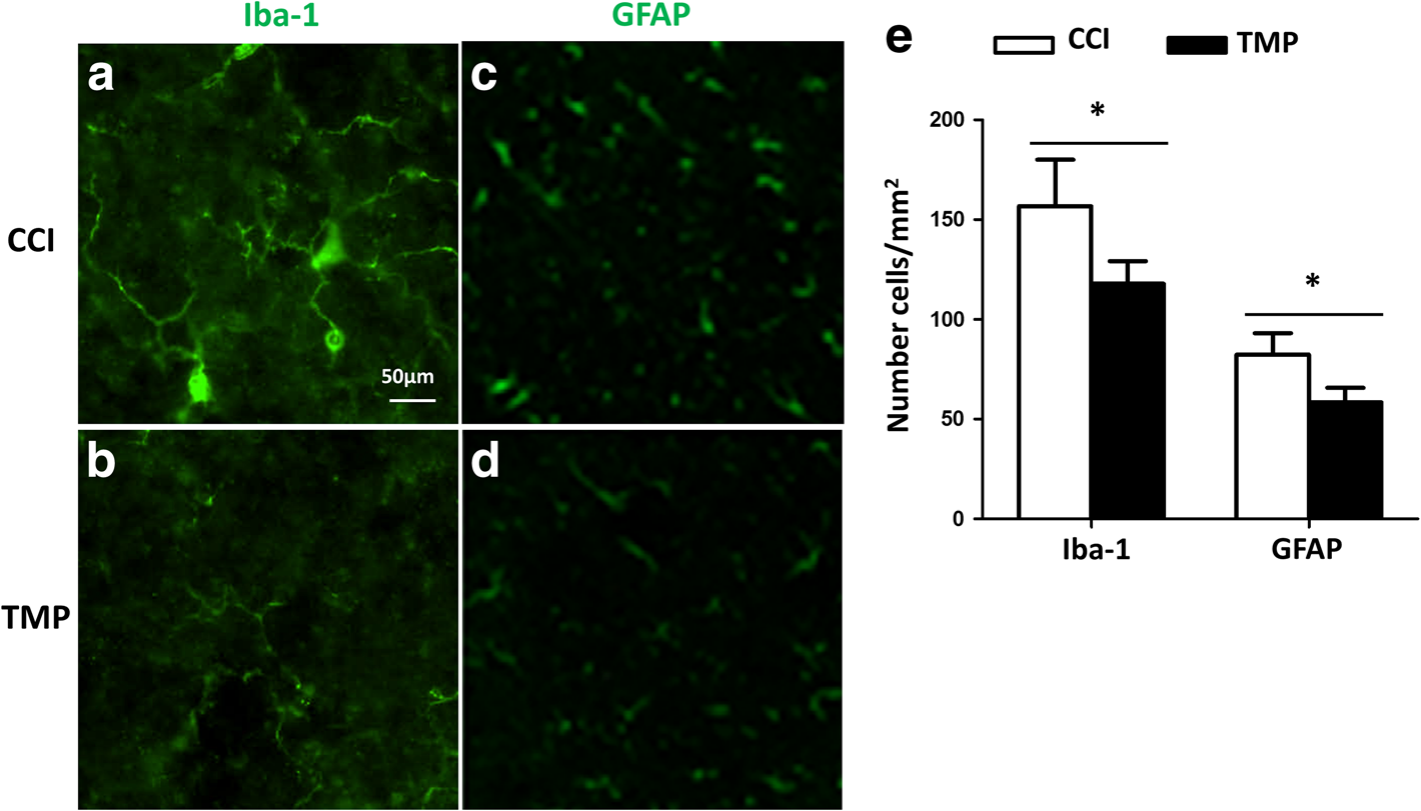
TMP reduced the activation of microglia and astrocytes in CCI mice. Ionized calcium-binding adaptor molecule-1 (Iba-1) is a marker in perilesional microglia/ macrophage cells, and GFAP, a marker for activated astrocytes, were immunostained with their antibodies 3 days after CCI surgery. (**a**, **b**) The levels of Iba-1^+^ microglia/macrophage cells and processes in CCI mice were attenuated by 4 mM TMP; (**c**, **d**) GFAP^+^ astrocytes and processes in CCI mice diminished when the mice were treated with 4 mM TMP; (**e**) Summary of Iba-1-positive microglia/macrophages and GFAP-positive astrocytes in CCI mice with and without the treatment of 4 mM TMP. *n* = 6 in each group. Data are presented as mean ± SEM values and representative of at least three independent experiments. Statistical analyses represent variations in experimental replicates. *P < 0.05; **P < 0.01

Glial cells are implicated in various neuropathic pain. We reasoned that TMP may reduce the activation of microglia and astrocytes in somatosensory cortex and medullary trigeminal zones of CCI mice. To confirm this notion, we did CCI surgery on mice, then, assigned them into two groups: one treated with saline, and another with 4 mM TMP. As expected, TMP treatment reduced the expression of Iba-1, a marker protein representing the activation of microglia (Fig. 3a, b), and attenuated the levels of GFAP, a marker protein expressed in the activated astrocytes (Fig. 3c, d). We counted cells expressing either Iba-1 or GFAP (Fig. 3e), and found that TMP reduced the number of these cells. Therefore, TMP treatment could alleviate the enhanced activation of microglia and astrocytes.

### TMP inhibited the expression of pro-inflammatory cytokines in CCI mice

**Fig. 4:**
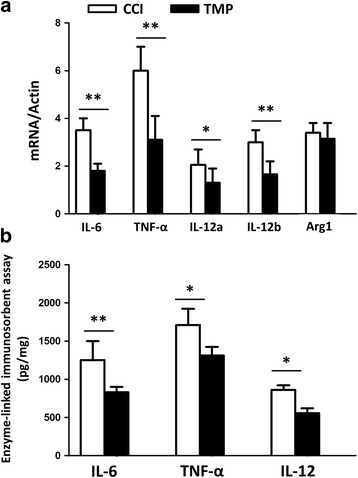
TMP lowered the level of pro-inflammatory cytokines in CCI mice. CCI mice were assigned into two groups: treated with or without TMP. Tissues were collected 3 days after surgery. (**a**) qRT-PCR analysis of the indicated genes in microglia of CCI mice. Data were presented as ratio of the levels of indicated mRNAs to those of Actin mRNA. (**b**) ELISA assay of the indicated cytokines in microglia. *n* = 6 in each group. Data were from at least three independent experiments, and are presented as mean ± SEM values. *P < 0.05; **P < 0.01

Microglia and astrocytes release numerous types of cytokines, which subsequently exacerbate the pathophysiology of neuropathic pain. Because TMP attenuated the activation of microglia and astrocytes, we next examined whether TMP also counteracts the release of cytokines from microglia and astrocytes (Fig. 4a, b). In Fig. 4a, qPCR assay showed that TMP significantly lowered the mRNA levels of pro-inflammatory cytokines, including IL-6, TNF-α,IL-12a, and IL-12b, but not Arg1, an anti-inflammatory factor gene. Similarly, the data from ELISA assay also showed less IL-6, TNF-α,and IL-12 in CCI + TMP mice, in comparison with CCI mice. These results indicated that TMP may have anti-inflammatory property in CCI mice.

### TMP regulated NF- κB pathway in microglia

**Fig. 5:**
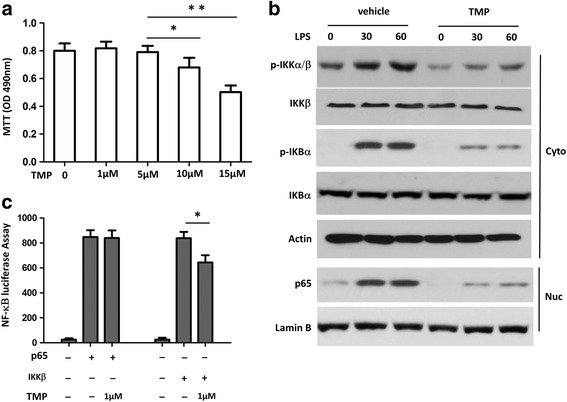
TMP attenuated the activation of NF-κB pathway by lipopolysaccharide exposure in microglia. (**a**) Cell viability was evaluated by the MTT assay. The effects of concentrations of TMP on cell viability were examined 24 h after plating of microglia. The data were collected from 6 different preparations. * *P* < 0.05, ** *P* < 0.001 vs. control. (**b**) Immunoblot analyses of phosphorylated (P-) IKKα/β and IKBα in cytoplasmic (Cyto) contents and p65 in nucleus (Nuc) extracts from microglia, stimulated with LPS for the indicated time periods with or without 5 μM TMP; (**c**) HEK293 cells (2 × 10^5^) were transfected with the IL-6 promoter luciferase plasmid and cotransfected with expression plasmid as indicated. Cells were incubated with 5 μM TMP for 2 h before harvest. Luciferase assays were performed 36 h after transfection. Data are representative of three independent experiments. *P < 0.05

To understand whether TMP interrupts NF-κB pathway commonly involved in the activation of microglia, we isolated microglia from the brains of neonatal mice (see Methods and Materials), and examined whether TMP alters the mobilization of NF-κB signaling pathway in response to lipopolysaccharide (LPS). Before we tested the effects of TMP on NF-κB signaling pathway, we examined the apoptosis of microglia in the presence of concentrations of TMP. MTT assay was used to detect viable cells. Decrease of optical density (OD) of MTT staining represents reduced viability of cells. As illustrated in Fig. 5a, 10 and 15 μM TMP enhanced apoptosis or death of microglia. Therefore, we chose 5 μM TMP in our experiments. We treated microglia with either vehicle or TMP for 24 h, and then tested the mobilization of NF-κB signaling pathway by 30 and 60 min incubation of LPS. We analyzed several molecules in NF-κB pathway, including IKKβ, IKBα, and P65. The former two are in the cytoplasm, while the latter one locates in the nucleus (Fig. 5b). The mobilization of NF-κB pathway starts from the phosphorylation, and subsequent degradation of IKBα, followed by the translocation of NF-κB from cytoplasm into nucleus. The phosphorylation of IKBα depends on the levels of IKKα/β. In vehicle-treated microglia, we observed that LPS time-dependently enhanced phosphorylation of IKKα/β and IKBα, and increased the levels of p65. In contrast, 24 h treatment with TMP significantly attenuated LPS effects. After entering the nucleus of the cell, NF-κB triggers the expression of various genes. To address whether reduction of p65 and phosphorylated IKKβ by TMP alters the expression of genes, such as IL-6, typically existing in microglia, we used luciferase assay. We transfected HEK293 cells with luciferase whose expression is driven by IL-6 promoter. As illustrated in Fig. 5c, co-transfection of either p65 or IKKβ promoted the expression of luciferase, interestingly, the presence of TMP did not alter the effect of p65, but inhibited the effect of IKKβ, on the expression of luciferase. These data suggest that the targets of TMP locate in the cytoplasm, instead of the nucleus.

## Discussion

In the present study, we successfully established TBI mouse models with CCI. As illustrated in Fig. 1a, the mice showed a significant decrease in periorbital threshold in comparison with sham mice. In CCI mice, we observed that the decrease of periorbital threshold reached the lowest levels at week 2. This phenomenon indicates that the development of pain behaviors might be secondary events, instead of immediate ones, following CCI. This time course is similar to the development of pain symptoms in TBI patients, which often takes weeks or months to manifest [21]. Interestingly, we observed that CCI on the right hemisphere led to the reduction of periorbital threshold on both sides. These data suggest that CCI on one hemisphere may affect pain processing circuits in both hemispheres. Although restraining the mouse in a narrow tube for periorbital mechanical threshold is a stress, but it may not interfere mechanical allodynia after CCI [15].

TMP is proved to effectively relieve pain symptoms induced by acute nociceptive stimulation, such as local injection of ATP, formalin or PGE2 [5], by burns [10], and by chronic constriction injury [8, 9]. We provide evidence showing that TMP significantly attenuated periorbital hypersensitivity following CCI. As illustrated in Fig. 1a, and b, TMP showed stronger inhibition on pain 3-4 weeks, than 1-2 weeks, after CCI, and even restored periorbital threshold to normal levels. These results suggest that TMP may differentially affect the development and recovery of periorbital hypersensitivity after CCI.

Periorbital skin is innervated by peripheral axons of the trigeminal ganglion, through which the nociceptive signals in periorbital region is relayed to trigeminal nucleus. Previous studies revealed that CCI modifies this relay pathway by upregulating CGRP, SP, and iNOS [13, 14, 16]. Importantly, both antagonizing these molecules and knocking-out of iNOS alleviate pain hypersensitivity in response to CCI [13]. In the present study, we observed that TMP reduced the levels of CGRP, SP, and iNOS in CCI mice, indicating that these molecules were also targeted by TMP, and could mediate the beneficial effects of TMP on periorbital pain.

Neuro-inflammation is a common aspect of pathophysiology in TBI models, including CCI [14, 22, 23]. It contains several steps of events and manifestations, such as, activation of microglia and astrocytes, and elevated synthesis and release of pro-inflammatory cytokines, and also enhanced mobilization of NF-κB signaling pathway. To counteract these alterations might be one of the major strategies to treat TBI. In this study, we have shown that TMP limits these pathophysiological features in CCI mice. This supports the notion that TMP might be a promising drug to relieve pain hypersensitivity in TBI.

First, TMP significantly reduced the levels of Iba-1 and GFAP, and also the number of activated microglia and astrocytes in the brain stem of CCI mice. Second, TMP led to a diminution of pro-inflammatory cytokines, such as, IL-6, TNF-α, IL-12, at both mRNA and protein levels. Third, TMP reversed the enhanced phosphorylation of IKBα and IKKα/β and expression of p65 after LPS treatment, suggesting an attenuated activation of NF-κB signaling pathway. Using luciferase assay in an in vitro system, we observed that TMP reduced cytoplasmic IKKβ-, but not nucleic p65-, induced enhancement of IL-6 expression, suggesting that the targets of TMP in modulating NF-κB signaling pathway may locate in cytoplasm, but not in nucleus. TMP-induced reduction of p65 in microglia may result from the tempered phosphorylation of IKB-α and translocation of NF-κB from cytoplasm into nucleus.

The detrimental outcomes of TBI include damage immediately or secondarily following primary injury [1, 14, 24]. In the present study, we examined pain behavior 1-4 weeks after CCI surgery, but analyzed most cellular and molecular events within 3 days after CCI. That is, we tested both immediate and secondary damage in the nervous system by CCI. From the time points examined, it seems that the cellular and molecular effects of TMP may not directly relate to its behavioral action. This suspicion tends to be supported by another observation that CGRP levels in CCI mice diminished starting from day 3 till day 14 after CCI. However, as illustrated in Fig. 2f, the levels of CGRP at day 1, 3, 7 in TMP-treated CCI mice were at the same levels at day 3, 7, 14 in CCI mice, correspondingly. It suggests that TMP was limiting CGRP levels starting very early after CCI surgery. This reaction pattern follows the possibility that the reversal of pain hypersensitivity by TMP seen in Fig. 1 might result from accumulated restriction of cellular and molecular events in Figs. 2- 5.

## Conclusions

In summary, the present study replicated a TBI pain model with CCI, and demonstrated that TMP relieved pain symptom at secondary phase in this model. Our cellular and molecular experiments at early phase of TBI support that TMP attenuated the activation of microglia and astrocytes, levels of SP, iNOS, and CGRP in trigeminal pathway, and levels of proinflammatory cytokines. The in vitro experiments showed that TMP inhibited LPS-induced cytoplasmic mobilization of NF-κB signaling pathway. The results support that TMP reversed pain hypersensitivity by limiting a series of cellular and molecular events in neuroinflammation occurred at the primary stage of TBI, could be a promising drug for TBI treatment. Of note, the limitation of our study is that only mouse CCI model of TBI was employed, which differs from human TBI. Future studies may focus on TBI animal model more relevant to human TBI.

## Abbreviations

CCI: Controlled cortical impact
iNOS: Nitric oxide synthase
SP: Substance P
TBI: Traumatic brain injury
TMP: Tetramethylpyrazine
